# Nephroprotective Role of Chrysophanol in Hypoxia/Reoxygenation-Induced Renal Cell Damage via Apoptosis, ER Stress, and Ferroptosis

**DOI:** 10.3390/biomedicines9091283

**Published:** 2021-09-21

**Authors:** Chih-Hung Lin, Han-Fang Tseng, Po-Chun Hsieh, Valeria Chiu, Ting-Yun Lin, Chou-Chin Lan, I-Shiang Tzeng, Huan-Nung Chao, Chia-Chen Hsu, Chan-Yen Kuo

**Affiliations:** 1 Department of Internal Medicine, Cathay General Hospital, Taipei 106, Taiwan; linchihhungjhsph@gmail.com; 2 School of Medicine, College of Medicine, Fu Jen Catholic University, New Taipei City 242, Taiwan; 3 Ph.D. Program in Nutrition and Food Science, Fu Jen Catholic University, New Taipei City 242, Taiwan; 4 Department of Anesthesiology, Taichung Veterans General Hospital, Taichung 407, Taiwan; spot1229@vghtc.gov.tw; 5 Department of Chinese Medicine, Taipei Tzu Chi Hospital, Buddhist Tzu Chi Medical Foundation, New Taipei City 231, Taiwan; pchsieh.tcm@gmail.com; 6 Division of Physical Medicine and Rehabilitation, Taipei Tzu Chi Hospital, Buddhist Tzu Chi Medical Foundation, New Taipei City 231, Taiwan; haydenbell28@gmail.com; 7 Department of Medicine, Tzu-Chi University, Hualien 970, Taiwan; water_h2o_6@hotmail.com (T.-Y.L.); bluescopy@yahoo.com.tw (C.-C.L.); 8 Division of Nephrology, Taipei Tzu Chi Hospital, Buddhist Tzu Chi Medical Foundation, New Taipei City 231, Taiwan; 9 Division of Pulmonary Medicine, Taipei Tzu Chi Hospital, Buddhist Tzu Chi Medical Foundation, New Taipei City 231, Taiwan; 10 Department of Research, Taipei Tzu Chi Hospital, Buddhist Tzu Chi Medical Foundation, New Taipei City 231, Taiwan; istzeng@gmail.com (I.-S.T.); because0517@gmail.com (C.-C.H.); 11 Department of Nephrology, Hanming Christian Hospital, Changhua City 500, Taiwan; hlc6885@gmail.com; 12 Department of Nursing, Cardinal Tien College of Healthcare and Management, New Taipei City 231, Taiwan

**Keywords:** chrysophanol, acute kidney injury (AKI), ER stress, ferroptosis

## Abstract

Acute kidney injury (AKI) is caused by hypoxia-reoxygenation (H/R), which is a kidney injury produced by a variety of causes, resulting in the remaining portion of the kidney function being unable to maintain the balance for performing the tasks of waste excretion metabolism, and electrolyte and acid-base balance. Many studies have reported the use of Chinese medicine to slow down the progression and alleviate the complications of chronic renal failure. Chrysophanol is a component of *Rheum officinale Baill*, a traditional Chinese medicine that has been clinically used to treat renal disease. We aimed to study the nephroprotective effect of chrysophanol on hypoxia/ reoxygenation (H/R)-induced cell damage. The results showed that chrysophanol prevented H/R-induced apoptosis via downregulation of cleaved Caspase-3, p-JNK, and Bax but upregulation of Bcl-2 expression. In contrast, chrysophanol attenuated H/R-induced endoplasmic reticulum (ER) stress via the downregulation of CHOP and p-IRE1α expression. Our data demonstrated that chrysophanol alleviated H/R-induced lipid ROS accumulation and ferroptosis. Therefore, we propose that chrysophanol may have a protective effect against AKI by regulating apoptosis, ER stress, and ferroptosis.

## 1. Introduction

It is well known that acute kidney injury (AKI) is a clinical syndrome characterized by a rapid loss of renal function that may further develop into chronic kidney disease (CKD) or even end-stage renal disease (ESRD) [1]. Many studies have reported the use of Chinese medicine to slow down the progression and alleviate the complications of acute renal failure, thereby improving quality of life [2,3]. In a cisplatin-induced AKI mouse model, wogonin reversed abrupt kidney dysfunction by substantially suppressing the increased levels of serum creatinine and blood urea nitrogen (BUN) to almost normal levels [4]. According to the literature on Chinese herbal intervention in AKI, Chinese herbs have been reported to play a critical role in the management of AKI by promoting repair and regeneration, enhancing extrarenal clearance of uremic toxins, and preventing progression to CKD [5,6]. Therefore, determining the best possible treatment may be a good strategy to attenuate or prevent renal injury in AKI.

Chrysophanol *is a natural anthraquinone*, also known as 1,8-dihydroxy-3-methyl-anthraquinone and chrysophanic acid, and is widely used in the food and pharmaceutical industries [7]. Moreover, chrysophanol has been reported to have various pharmacological properties, including antidiabetic, anticancer, neuroprotective, hepatoprotective, anti-ulcer, anti-inflammatory, antiviral, antifungal, and antibacterial activities [8]; however, the effect of chrysophanol on AKI remains unclear. AKI is a major cause of renal ischemia–reperfusion (I/R) or hypoxia-reperfusion or hypoxia-reoxygenation (H/R), as demonstrated in cell lines and mouse models [9,10]. Accumulating evidence indicates that proximal tubular cells (HK-2 cells) are sensitive to renal I/R [11,12,13].

Endoplasmic reticulum (ER) stress is the major cause of renal cell damage during the progression of AKI by renal I/R [14]. Furthermore, excessive reactive oxygen species (ROS) that cause oxidative stress may contribute to the increased susceptibility of the aging kidney in prolonging ER stress-induced acute injury [15]. Mounting evidence indicates that ER stress contributes to glomerular and tubular damage in patients with acute and chronic kidney disease [16]. Therefore, modulation and normalization of ER stress in kidney cells using pharmacological agents may be a promising therapeutic approach for preventing the progression of kidney disease [17]. On the other hand, an increasing number of studies have significantly implicated ferroptosis, a unique type of regulated cell death that is characterized by a large amount of iron accumulation and lipid peroxidation during the cell-death process, in the development of acute kidney disease [18,19,20]. Altogether, targeting the homeostasis of ferroptosis may provide a new target for therapeutic intervention in AKI [21]. Shu et al. reported that I/R led to kidney injury via the underlying mechanism of ER stress as indicated by the increased expression of the C/EBP homologous protein (CHOP). Additionally, inhibition of ER stress prevents renal tubular epithelial cell apoptosis, inflammation, and autophagy caused by I/R [22].

Recent studies have revealed an emerging role of ferroptosis in the pathophysiological processes of AKI, which can eventually lead to acute renal failure (ARF) [19,23,24]. Yang and Stockwell suggested that the downregulation of GPX4 may be more sensitive to ferroptosis, whereas inhibition of ferroptosis was detected upon the upregulation of GPX4 in vitro [25]. Furthermore, SLC7A11 is a key regulator of ferroptosis [18]. It has also been reported that inhibiting SLC7A11 activity induces ROS accumulation and triggers ferroptosis [26].

In the present study, we investigated whether chrysophanol attenuated cell death in HK-2 cells exposed to H/R injury and exerted renal protection via modulation of apoptosis, ER stress, and ferroptosis. 

## 2. Materials and Methods

### 2.1. Reagents and Antibodies

Chrysophanol (CAS 481-74-3) was purchased from Sigma (St. Louis, MO, USA). The chrysophanol was solved in DMSO (C6164, Sigma, St. Louis, MO, USA) for 30 mM. The cell proliferation reagents WST-1 and RNase A were obtained from Roche Applied Sciences (Mannheim, Germany). Deferoxamine mesylate salt (DFO, D9533, Sigma, St. Louis, MO, USA). The DFO was solved in phosphate-buffered saline (PBS, P5493, Sigma, St. Louis, MO, USA) for 0.1 M. The same volume of vehicle (DMSO or PBS) was added to the controls in this study.

The antibodies used for immunofluorescence staining and Western blotting were as follows: rabbit polyclonal antibodies against cleaved Caspase-3 (Cell Signaling Technology, Danvers, MA, USA), β-actin (Cell Signaling, Danvers, MA, USA), CHOP (ABclonal, Woburn, MA, USA), p-IRE1α (ABclonal, Woburn, MA, USA), IRE1α (ABclonal, Woburn, MA, USA), p-JNK (ABclonal, Woburn, MA, USA), JNK (ABclonal, Woburn, MA, USA), IL-6 (ABclonal, Woburn, MA, USA), GAPDH (Cell Signaling, Danvers, MA, USA), p65 (Cell Signaling Technology, Danvers, MA, USA), and fibrillin (Cell Signaling, Danvers, MA, USA).

### 2.2. Cell Culture and Hypoxia/Reoxygenation (H/R) Conditions

HK-2 cells (human renal proximal tubular epithelial cells) were purchased from BCRC (Bioresource Collection and Research Center, Hsinchu, Taiwan) and cultured in T-75 flasks (Corning, Corning, NY, USA) in DMEM/Ham’s F12 (Gibco, New York, NY, USA) supplemented with 10% heat-inactivated fetal bovine serum, 25 mM D-glucose, 2 mM L-glutamine, 1 mM sodium pyruvate, and penicillin-streptomycin (50 U/mL; Sigma, St. Louis, MO, USA) at 37 °C in 5% CO_2_/95% air. The culture medium was replaced with fresh medium on alternate days. Once the cells reached 60–70% confluence, they were trypsinized for subsequent experiments. Mycoplasma test results were negative.

Hypoxia/reoxygenation conditions were created using a previously described method with some modifications [27,28]. Briefly, cells were grown on 6- or 24-well plastic dishes in a hypoxia chamber and equilibrated for 30 min with humidified gas containing 1% oxygen, 5% CO_2_, and 94% nitrogen (Hypoxic incubator APM-30D, Astec, Tokyo, Japan). The cell lines were maintained under hypoxic conditions for 15 h and then additionally incubated under normoxic conditions for another 2 h. The normal group consisted of cells grown under normal (21%) oxygen conditions for the same duration.

### 2.3. Cell Viability Assay

Cell viability was measured using the WST-1 assay. Cells were seeded at a density of 5 × 10^4^ cells/mL in 24-well plates and cultured in phenol red-free DMEM containing 0.5% heat-inactivated FBS for 24 h. Then, the cells were incubated with the indicated concentrations of chrysophanol (30 μM) for 24 h. The WST-1 reagent was then added to the medium and the cells were incubated at 37 °C for 2 h. The absorbance was measured at 450 nm using a microplate reader (Bio-Rad, Hercules, CA, USA).

### 2.4. Western Blotting

The cells 1 × 10^6^ were harvested and lysed according to a protocol described in our previous study [29]. Briefly, cells were collected, washed three times with PBS and lysed using RIPA lysis buffer (Pierce, Rockford, IL, USA), containing 1% Sigma protease cocktail, for 30 min at 4 °C. The lysates were centrifuged at 10,000× *g* at 4 °C to obtain solubilized cellular proteins. The supernatant protein concentration was measured using a bicinchoninic acid (BCA) protein assay (Pierce, Rockford, IL, USA). Proteins were separated by 8%, 10% or 12% SDS-PAGE and electro-transferred onto a polyvinylidene fluoride membrane. Blots were probed with specific primary antibodies and followed by HRP-conjugated goat anti-rabbit IgG (1:3000–1:5000) or HRP-conjugated goat anti-mouse IgG (1:5000) (Zymed, San Francisco, CA, USA). After washing with PBS containing 0.5% Tween-20, peroxidase activity was assessed using enhanced chemiluminescence (ECL; PerkinElmer Life Science, Hopkinton, MA, USA). The same membrane was re-probed with a monoclonal antibody directed against GAPDH or β-actin as a loading control (1:5000; GeneTex, Irvine, CA, USA). The intensities of the reaction bands were analyzed with the UVP Biospectrum (UVP, LLC Upland, CA, USA). The primary antibodies used are mentioned in Section 2.1 (Reagents and antibodies).

### 2.5. Lipid ROS Detection

Cells were incubated with 2 µM C11-BODIPY 581/591 (Thermo Fisher Scientific, Waltham, MA, USA) in culture medium for 1 h and then washed with phosphate-buffered saline (PBS). After trypsinization, cells were collected and processed for flow cytometry (BD Biosciences, San Jose, CA, USA) at an excitation wavelength of 488 nm and an emission wavelength of 517–527 nm.

### 2.6. Nuclear Fraction Extraction

The nuclear fraction was extracted from 1 × 10^6^ HK-2 cells according to the protocol of a previous study [30]. Briefly, nuclear fraction was extracted from cells. The cells were collected and resuspended in a hypotonic buffer (10 mM HEPES, pH 7.9; 10 mM KCl; 1.5 mM MgCl_2_; 0.2 mM PMSF; 20 μg/mL aprotinin; 0.5 mM DTT; and 0.5% NP-40) on ice for 15 min. After centrifuging at 6000× *g* for 15 min at 4 °C, the pellet was collected and then washed with basal buffer (hypotonic buffer without 0.5% NP-40). After centrifuging again at 6000× *g* for 15 min at 4 °C, the pellet was collected and resuspended in a hypertonic buffer (20 mM HEPES, pH 7.9; 400 mM KCl; 1.5 mM MgCl_2_; 0.2 mM PMSF; 20 μg/mL aprotinin; 0.5 mM DTT; 0.2 mM EDTA; 10% glycerol) at room temperature for 30 min. After centrifuging at 10,000× *g* for 30 min at 4 °C, the nuclear fraction contained in the supernatant was collected.

### 2.7. Immunofluorescence Staining

The cells were fixed in 3% formaldehyde at room temperature for 30 min, blocked with PBS containing 3% FBS at room temperature for 1 h, and incubated with anti-p65 antibody and β-catenin at 4 °C for 16 h, followed by incubation with FITC-labeled secondary antibody (Jackson ImmunoResearch Laboratories, West Grove, PA, USA) at room temperature for 1 h. The nuclei were stained with diamidino-2-phenylindole (DAPI, Molecular Probes) at room temperature for 15 min, which is a DNA groove-binding dye, and examined using a Leica TCS SP5 laser scanning microscope (Leica, Bensheim, Germany).

### 2.8. Statistical Analyses

All data were analyzed using one-way or two-way analysis of variance (ANOVA). Differences were considered statistically significant when the *p*-values were less than 0.05 (* *p* < 0.05, ** *p* < 0.01).

## 3. Results

### 3.1. Chrysophanol Attenuated H/R-Induced Cell Death via Apoptosis

**Figure 1:**
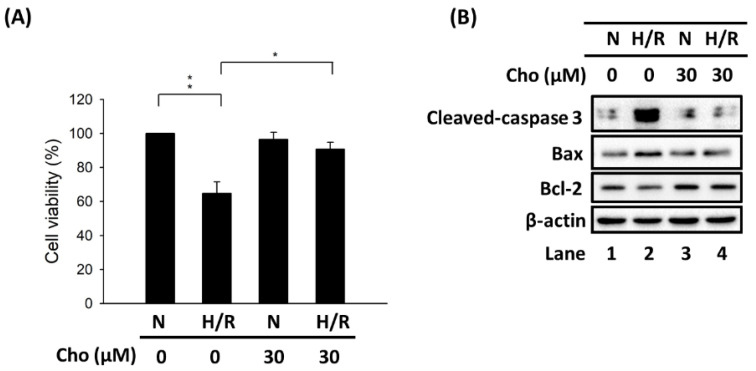
Chrysophanol alleviated H/R-induced cell viability. (**A**) Cell viability was determined using WST-1 assays for the indicated groups of cells. All data are presented as mean ± SD. n = 3. * *p* < 0.05. ** *p* < 0.01. (**B**) Changes in the expression of cleaved-caspase 3, Bax and Bcl-2. β-actin was used as an internal control. Cho: Chrysophanol. H/R: Hypoxia/reoxygenation. N: Normoxia.

H/R significantly induced cell death in HK-2 cells, according to the cell viability analysis (Figure 1A). To determine the effect of chrysophanol on cell survival, cell viability was determined in cells treated with 30 μM chrysophanol prior to incubation under H/R conditions. Results showed that chrysophanol significantly alleviated H/R-induced cell viability (Figure 1A). To further study whether chrysophanol attenuated H/R-induced cell death via apoptosis, we detected the expression of cleaved Caspase-3, Bax, and Bcl-2 in the presence of 30 μM chrysophanol under H/R conditions. The results showed a decrease in cleaved Caspase-3 and Bax but an increase in Bcl-2 was observed in the presence of 30 μM chrysophanol under H/R conditions (Figure 1B, lane 2 vs. lane 4). These results indicated that chrysophanol attenuated H/R-induced cell death via apoptosis.

### 3.2. Chrysophanol Regulated ER Stress and Ferroptosis Pathway under H/R Conditions

**Figure 2:**
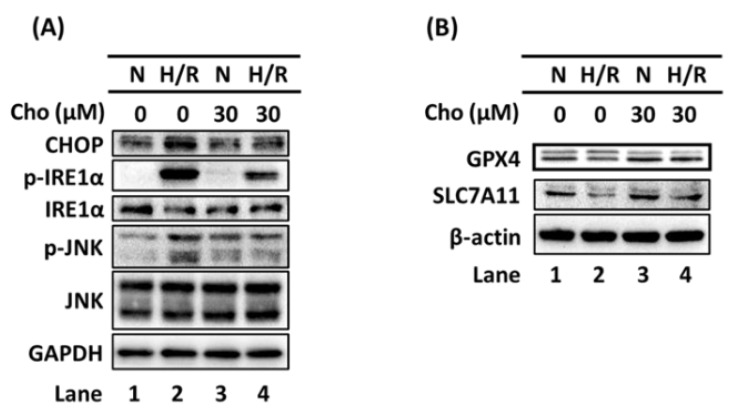
Chrysophanol alleviated H/R-induced ER stress and ferroptosis. (**A**) Changes in the expression of p-IRE1α. GAPDH was used as an internal control. (**B**) Changes in the expression of GPX4 and SLC7A11. β-actin was used as an internal control. Cho: Chrysophanol. H/R: Hypoxia/reoxigenation. N: Normoxia. n = 3.

To investigate the effect of chrysophanol on H/R-induced ER stress, we used Western blotting to examine the expression of CHOP and phosphorylated phosho-IRE1α (p-IRE1α) in the presence and absence of chrysophanol under H/R conditions. The results indicated that H/R increased the expression of CHOP and p-IRE1α (Figure 2A, lane 1 vs. lane 2). On the other hand, chrysophanol reversed the upregulation of CHOP and p-IRE1α under H/R conditions (Figure 2A, lane 2 vs. lane 4).

**Figure 3:**
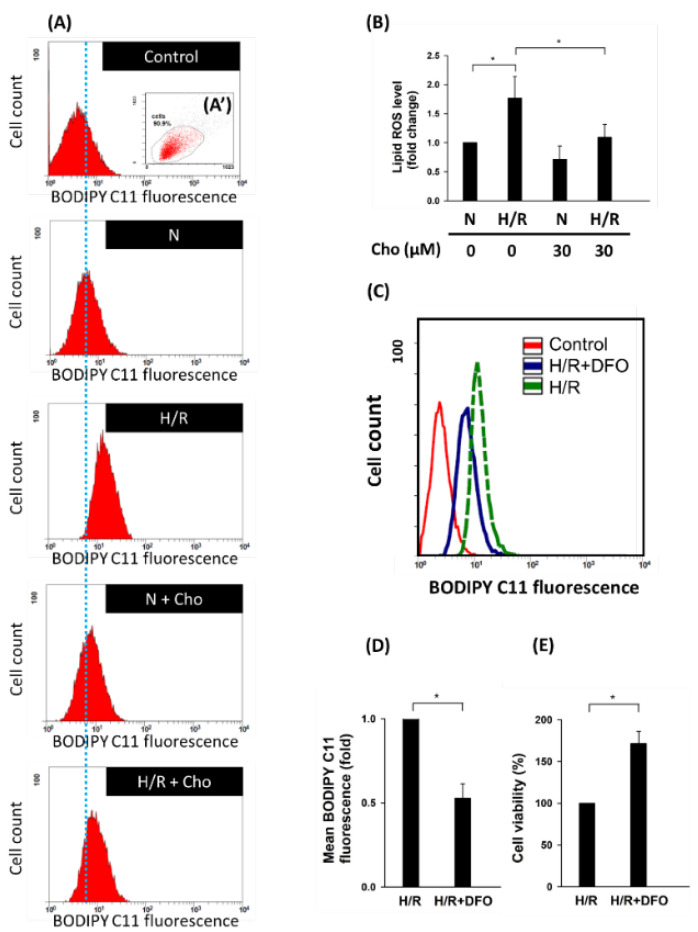
Chrysophanol reversed H/R-induced lipid ROS production. (**A**) Changes in cellular lipid ROS levels of control (without C11-BODIPY staining), N (cells grown in normal oxygen conditions without chrysophanol treatment), H/R (cells cultured in hypoxia/reoxygenation conditions, as described in Section 2), N + Cho (cells grown in normal oxygen conditions with 30 μM chrysophanol treatment), and H/R + Cho (cells cultured under hypoxia/reoxygenation conditions with 30 μM chrysophanol treatment) groups. **(A)** Cells were selected based on morphology. (**B**) ROS generation is expressed as mean fluorescence intensity. (**C**) Changes in cellular lipid ROS levels of control (without C11-BODIPY staining). H/R + DFO (cells cultured under hypoxia/reoxygenation conditions with 100 μM Desferal treatment for 1 h) groups (**D**) Lipid ROS generation is expressed as mean fluorescence intensity. (**E**) Cell viability was determined using WST-1 assays for the indicated groups of cells. Error bars represent the standard deviation from three independent replicates. n = 3. * *p* < 0.05.

In this study, results showed that H/R increased the expression of GPX4 and SLC7A11 (Figure 2B, lane 1 vs. lane 2). On the other hand, chrysophanol reversed the upregulation of GPX4 and SLC7A11 under H/R conditions (Figure 2B, lane 2 vs. lane 4). To further confirm the anti-ferroptotic effect of chrysophanol under H/R conditions, we measured the levels of lipid ROS (Figure 3). To further verify the role of iron under H/R conditions, we detected the levels of lipid ROS and cell viability in presence of DFO (iron chelator). Results showed that the DFO attenuated the increase (Figure 3C,D) and the decrease in cell viability under H/R conditions (Figure 3E). Taken together, we suggest that chrysophanol regulates ER stress and the ferroptosis pathway under H/R conditions.

### 3.3. Chrysophanol Treatment Attenuated p-JNK Expression and NF-κB Nuclear Translocation under H/R Conditions

**Figure 4:**
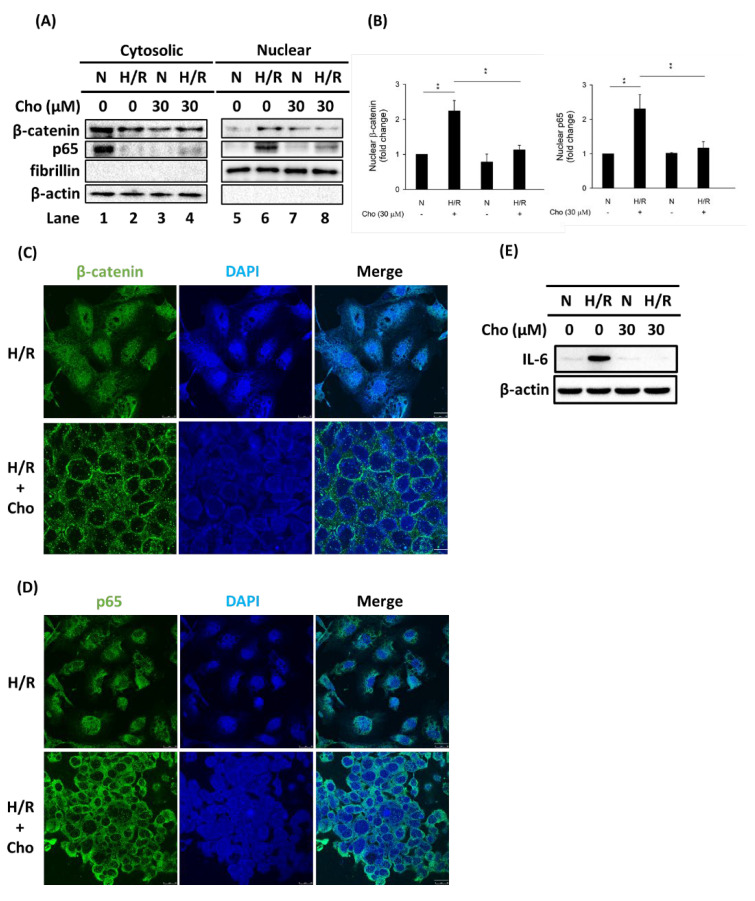
Chrysophanol reversed H/R-induced nuclear translocation of β-catenin and p65. (**A**) Cytosolic and nuclear fractions were isolated as described in “materials and methods”. Western blot analysis was performed to detect the subcellular localization of β-catenin and NF-κB using an antibody against the β-catenin and NF-κB subunit p65. β-actin was used as a cytosolic marker and fibrillarin as a nuclear marker. (**B**) The expression of β-catenin and nuclear p65 (nuclear p65) was quantified using ImageJ. All data are presented as the mean ± SD. ** *p* < 0.01. Immunofluorescence micrographs showed the distribution of β-catenin (**C**) and p65 (**D**). The β-catenin (**C**) and p65 (**D**) signal was intensely localized to the nucleus in HK-2 cells under H/R conditions (H/R) but was located in the cytoplasm under H/R conditions in the presence of 30 μM chrysophanol (H/R + Cho). The nuclei were counterstained with DAPI. Bar = 25 μm. (**E**) Chrysophanol alleviated the increasing in the IL-6 expression under H/R conditions. n = 3.

Our results demonstrated that H/R increased the expression of p-JNK (Figure 2A, lane 1 vs. lane 2) and NF-κB nuclear translocation (Figure 4A, lane 5 vs. lane 6). On the other hand, we also found that chrysophanol reversed the increase in p-JNK expression (Figure 2A, lane 2 vs. lane 4) and the expression of NF-κB nuclear translocation under H/R conditions (Figure 4A, lane 6 vs. lane 8). The quantitative results were shown in Figure 4B. Additionally, to further confirm the effect of chrysophanol on β-catenin and NF-κB p65 nuclear translocation under H/R conditions, we performed immunofluorescence staining (Figure 4C,D and Supplementary Materials Figure S1). Moreover, results also demonstrated that chrysophanol alleviated the increase in IL-6 expression under H/R conditions (Figure 4E). These results indicated that chrysophanol treatment attenuated p-JNK expression and NF-κB nuclear translocation under H/R conditions.

## 4. Discussion

In this study, we proposed that chrysophanol has a nephroprotective effect on renal cell damage caused by H/R. Additionally, we also suggested that apoptosis, ER stress, and ferroptosis, which are involved in H/R-induced cell death, were reversed in the presence of chrysophanol. Havasi and Borkan demonstrated that the improvement of renal cell death in AKI generated new therapeutic targets [31]. Interestingly, proximal tubular epithelial cells were reported to be highly susceptible to apoptosis, and injury at this site contributes to kidney failure [31]. Human renal proximal tubular cells (HK-2) were established as a cell model of hypoxia-reoxygenation (H/R) injury to mimic acute renal I/R injury [32], as well as our findings (Figure 1). Traditional medicines often use multi-component extracts of natural products that may be developed as therapeutic strategies to treat AKI due to their multitarget potential and established biosafety [33]. *Rhus verniciflua* Stokes extract was reported to prevent the progression of AKI via modulation of the Nrf2/antioxidant enzyme pathway, using in vivo and in vitro I/R injury (IRI)-induced AKI models [34]. Similarly, *Taraxacum officinale* has a protective effect on H/R-induced AKI via inhibition of oxidative stress, inflammation, and apoptosis in the extracellular signal-regulated kinase (ERK) and c-Jun NH2-terminal kinase (JNK) of the mitogen-activated protein kinase (MAPK) signaling pathways in vivo and in vitro [35]. Accumulating evidence has revealed that natural products or herbs may have potential for use in the clinical treatment of renal H/R injury [36,37,38], but resveratrol did not protect against H/R-induced AKI in vivo [39].

It is well known that regulating ER stress in kidney cells may provide a therapeutic target in AKI that is caused by H/R [14,16,40,41]. As shown in Figure 2A, H/R-induced ER stress was attenuated by chrysophanol treatment. Consistent with our findings, Huaier extract was found to downregulate the expression of CHOP and Bip in a thapsigargin-induced ER stress model of HK-2 cells [42]; the same result was also observed in other studies [27]. On the other hand, ferroptosis is well known to play a critical role in the pathology of AKI [19]. Quercetin (QCT) was reported to inhibit ferroptosis, but not apoptosis, necrosis, or autophagy, in renal proximal tubular epithelial cells and ameliorate AKI induced by I/R or folic acid (FA) [43]. In this study, chrysophanol attenuated H/R-induced ferroptosis via the regulation of GPX4 and SLC7A11, as seen on Western blot (Figure 2B) and BODIPY C11 fluorescence staining (Figure 3) in HK-2 cells, similar to the results obtained in other studies [44,45]. Therefore, chrysophanol ameliorated renal cell injury with H/R, perhaps by inhibiting ferroptosis. Chrysophanol may represent a novel treatment option that improves recovery from H/R-induced renal tubular cell injury by targeting ferroptosis. It is also possible that chrysophanol acts as an antioxidant, which may consequently increase the antioxidative capacity of the cells and elevate the expression of GXP4 and SLC7A11.

NF-κB signaling was reported to play a major role in the regulation of kidney injury [46]. Tubular injury was rescued by inhibiting NF-κB signaling in the renal tubular epithelium in vivo [47]. In addition, apoptotic tubular cell death was determined via downregulation of the Wnt/β-catenin dependent pathway by Dickkopf-3 in proteinuric nephropathy [48]. However, the conflicting role of the Wnt/β-catenin signaling pathway in kidney diseases and kidney injury repair or regeneration is determined by whether kidney structure and function are “reversible” or “irreversible” after injury. Therefore, further elucidation of the mechanisms by which Wnt/β-catenin acts in AKI and CKD may offer new therapeutic options for patients with various kidney diseases [49].

Cisplatin is one of the most widely used chemotherapeutic agents for treating solid tumors, but the administration of cisplatin causes tubular cell injury and AKI [50,51]. Maltol (3-hydroxy-2-methyl-4-pyrone), found in baked products as well as red ginseng root, coffee, chicory, soybeans, bread crusts, and caramelized foods [52], was reported to serve as a valuable potential drug to prevent cisplatin-induced nephrotoxicity [53]. Ridzuan et al. summarized several studies reporting that natural products possess potent antioxidant and anti-inflammatory medicinal properties, and they can be safely used as a supplementary regime or during combination therapy against cisplatin-induced nephrotoxicity [54]. In a future study, we will investigate whether chrysophanol alleviates cisplatin-induced nephrotoxicity.

## 5. Conclusions

**Figure 5:**
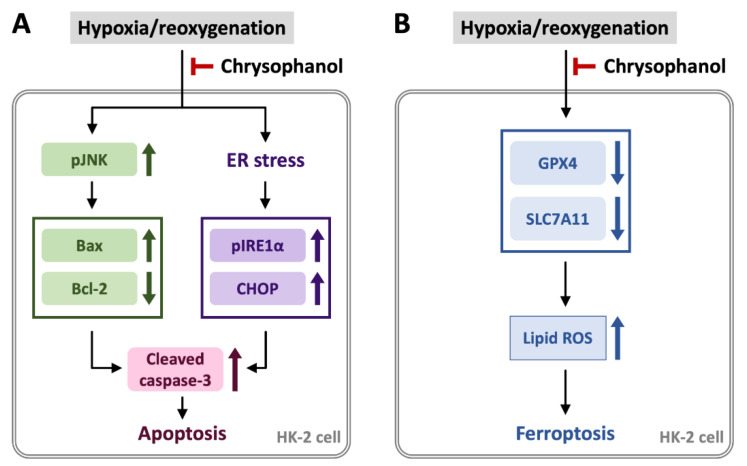
A summary diagram outlining the working mechanisms of chrysophanol in HK-2 cells under H/R conditions. (**A**) H/R increased apoptosis via upregulation of p-JNK, Bax, down-regulation of Bcl-2 and ER stress via upregulation of p-IRE1α/CHOP. Moreover, chrysophanol prevented apoptosis and ER stress by reversing the expression of indicated molecules. (**B**) H/R triggers ferroptosis via downregulation of GPX4 and SLC7A11, as well as lipid ROS overproduction. (**B**) Chrysophanol prevents H/R-induced ferroptosis via upregulation of GPX4 and SLC7A11, as well as decreasing lipid ROS accumulation.

In summary, the present study demonstrated that chrysophanol prevented H/R-induced cell death via apoptosis by increasing the expression of Bax, and p-JNK but decreasing the expression of Bcl-2, as well as prolonging ER stress by increasing the expression of CHOP and p-IRE1α, finally increasing the expression of cleaved Caspase-3 (Figure 5A). Additionally, chrysophanol attenuated the apoptosis via inhibition of apoptotic molecules, triggering the expression of anti-apoptotic molecules and inhibition of ER stress (Figure 5A). We also suggested that H/R induced HK-2 cell death by triggering ferroptosis via lipid ROS accumulation and downregulation of anti-ferroptotic molecules, GPX4 and SLC7A11 (Figure 5B). On the other hand, chrysophanol showed potential anti-ferroptotic effects in HK-2 cells under H/R conditions via upregulation of GPX4 and SCL7A11 and alleviating lipid ROS accumulation (Figure 5B)

## Data Availability

The original data used to support the findings of this study are included in this article.

## Supplementary Materials

The following are available online at https://www.mdpi.com/article/10.3390/biomedicines9091283/s1, Figure S1: Effect of chrysophanol on nuclear translocation of β-catenin and p65 under normoxia.
