# Supplementary material for: Nephroprotective Role of Chrysophanol in Hypoxia/Reoxygenation-Induced Renal Cell Damage via Apoptosis, ER Stress, and Ferroptosis
Source: Biomedicines. 2021 Sep 21;9(9):1283. doi: 10.3390/biomedicines9091283 (PMC8467645; doi:10.3390/biomedicines9091283)
Supplement: Supplementary file 1 [file biomedicines-09-01283-s001.zip › biomedicines-1355819-supplementary.pdf]

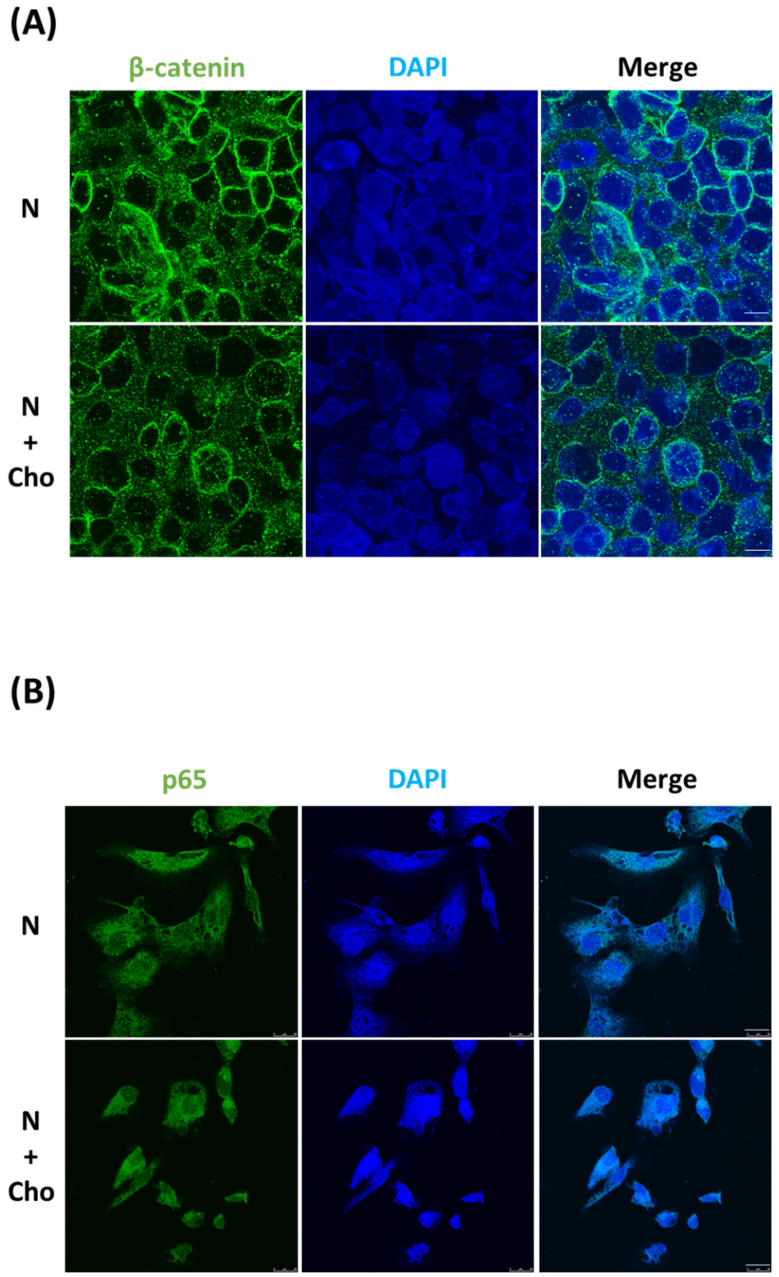

Figure S1. Effect of chrysophanol on nuclear translocation of β-catenin and p65 under normoxia. Immunofluorescence micrographs showed the distribution of β-catenin (A) and p65 (B). The β-catenin (A) and p65 (B) signal was intensely localized to the cytoplasm both in HK-2 cells under normoxia condition (N) and normoxia conditions in the presence of 30 μM chrysophanol (N+ Cho). The nuclei were counterstained with DAPI. Bar = 25 μm.
